# BH3-Only Protein BIM Mediates Heat Shock-Induced Apoptosis

**DOI:** 10.1371/journal.pone.0084388

**Published:** 2014-01-10

**Authors:** Indra M. Mahajan, Miao-Der Chen, Israel Muro, John D. Robertson, Casey W. Wright, Shawn B. Bratton

**Affiliations:** 1 The University of Texas MD Anderson Cancer Center, Science Park, Department of Molecular Carcinogenesis, Smithville, Texas, United States of America; 2 Division of Pharmacology and Toxicology, College of Pharmacy, The University of Texas at Austin, Austin, Texas, United States of America; 3 Department of Pharmacology, Toxicology and Therapeutics, University of Kansas Medical Center, Kansas City, Kansas, United States of America; Roswell Park Cancer Institute, United States of America

## Abstract

Acute heat shock can induce apoptosis through a canonical pathway involving the upstream activation of caspase-2, followed by BID cleavage and stimulation of the intrinsic pathway. Herein, we report that the BH3-only protein BIM, rather than BID, is essential to heat shock-induced cell death. We observed that BIM-deficient cells were highly resistant to heat shock, exhibiting short and long-term survival equivalent to *Bax^−/−^Bak^−/−^* cells and better than either *Bid^−/−^* or dominant-negative caspase-9-expressing cells. Only *Bim^−/−^* and *Bax^−/−^Bak^−/−^* cells exhibited resistance to mitochondrial outer membrane permeabilization and loss of mitochondrial inner membrane potential. Moreover, while dimerized caspase-2 failed to induce apoptosis in *Bid^−/−^* cells, it readily did so in *Bim^−/−^* cells, implying that caspase-2 kills exclusively through BID, not BIM. Finally, BIM reportedly associates with MCL-1 following heat shock, and *Mcl-1^−/−^* cells were indeed sensitized to heat shock-induced apoptosis. However, pharmacological inhibition of BCL-2 and BCL-X_L_ with ABT-737 also sensitized cells to heat shock, most likely through liberation of BIM. Thus, BIM mediates heat shock-induced apoptosis through a BAX/BAK-dependent pathway that is antagonized by antiapoptotic BCL-2 family members.

## Introduction

Apoptosis is an evolutionarily-conserved programmed form of cell death that involves the activation of caspases (cysteine proteases) [1]. These proteases are typically activated in response to stimulation of cell-surface death receptors, such as Fas/CD95, or in response to stressful stimuli, such as oncogene activation, DNA damage, growth factor withdrawal, ER stress, etc. [2]. In the latter instances, stress activates the so-called *intrinsic apoptosis pathway*, which generally involves the activation of pro-apoptotic BCL-2 family members. BH3-only proteins, such as BID, BIM, PUMA, BAD, and NOXA, serve as cellular sentinels that are activated in response to distinct types of stress. These BH3-only proteins subsequently activate the multidomain proapoptotic family members, BAX and BAK, which are often restrained by the antiapoptotic BCL-2 family members, BCL-2, BCL-X_L_, and/or MCL-1 [3]–[5]. How BH3-only family members activate BAX and BAK remains controversial, but BID, BIM, and PUMA are thought to directly activate BAX and BAK, whereas BAD, NOXA, and other BH3-only family members indirectly activate BAX and BAK (or contribute to their activation) through neutralization of the antiapoptotic family members [6]–[13].

Once activated, BAX inserts into the outer mitochondrial membrane, and both BAX and BAK oligomerize into pores that permeabilize the membrane and facilitate the release of intermembrane space proteins, such as cytochrome c (cyt c), into the cytoplasm. Cyt c then binds to apoptotic protease-activating factor 1 (Apaf-1) and triggers a (d)ATP-dependent conformational change in Apaf-1 that results in its oligomerization into a heptameric caspase-activating complex, known as the Apaf-1 apoptosome [14]. Finally, the apoptosome sequentially recruits and activates the initiator caspase-9 and the effector caspase-3, the latter of which targets >800 cellular substrates for proteolytic cleavage. Thus, cells utilize various BH3-only family members to integrate a variety of cellular stressors, all of which induce mitochondrial outer membrane permeabilization (MOMP), apoptosome assembly, caspase activation, and cell death [2].

BID is unique among the BH3-only family members in that it is activated through caspase cleavage, most notably by caspase-8, which allows death receptors to engage the intrinsic pathway. Interestingly, caspase-2 has also been shown to act upstream of mitochondria, particularly when overexpressed, and engages the intrinsic pathway through cleavage of BID [15]–[22]. However, assigning a definitive role for caspase-2 in stress-induced apoptosis *per se* has been problematic. Results from numerous studies have suggested that caspase-2 is either critical for DNA damaged-induced apoptosis or irrelevant to this response [23]. Caspase-2 knockout mice develop normally, aside from an increase in oocytes, but when crossed with the Eµ-myc or MMTV/c-neu mouse models, they develop significantly more lymphomas and mammary tumors, respectively, indicating a putative role for caspase-2 as a tumor suppressor [24]–[26].

Recently, caspase-2 has been shown to play an important role in cell death induced by microtubule disruption and heat shock [18], [21], [27], [28]. Indeed, Green and colleagues have shown that caspase-2 forms a complex with its adapter protein RAIDD, early following heat shock, and can be trapped with a biotinylated version of the polycaspase inhibitor zVAD-fmk [21], [28]. They also find that BID and BAX/BAK-deficient cells are resistant to caspase-2 and heat shock-induced apoptosis [18]. In the present study, we provide strong evidence that BIM mediates heat shock-induced apoptosis through a BAX/BAK-dependent pathway and that the caspase-2-BID pathway likely functions as either a parallel pathway in some cell-types or as part of an important amplification loop to enhance cell death, particularly at lower temperatures or decreased exposures.

## Results

### Bim is essential for heat shock-induced cell death

**Figure 1:**
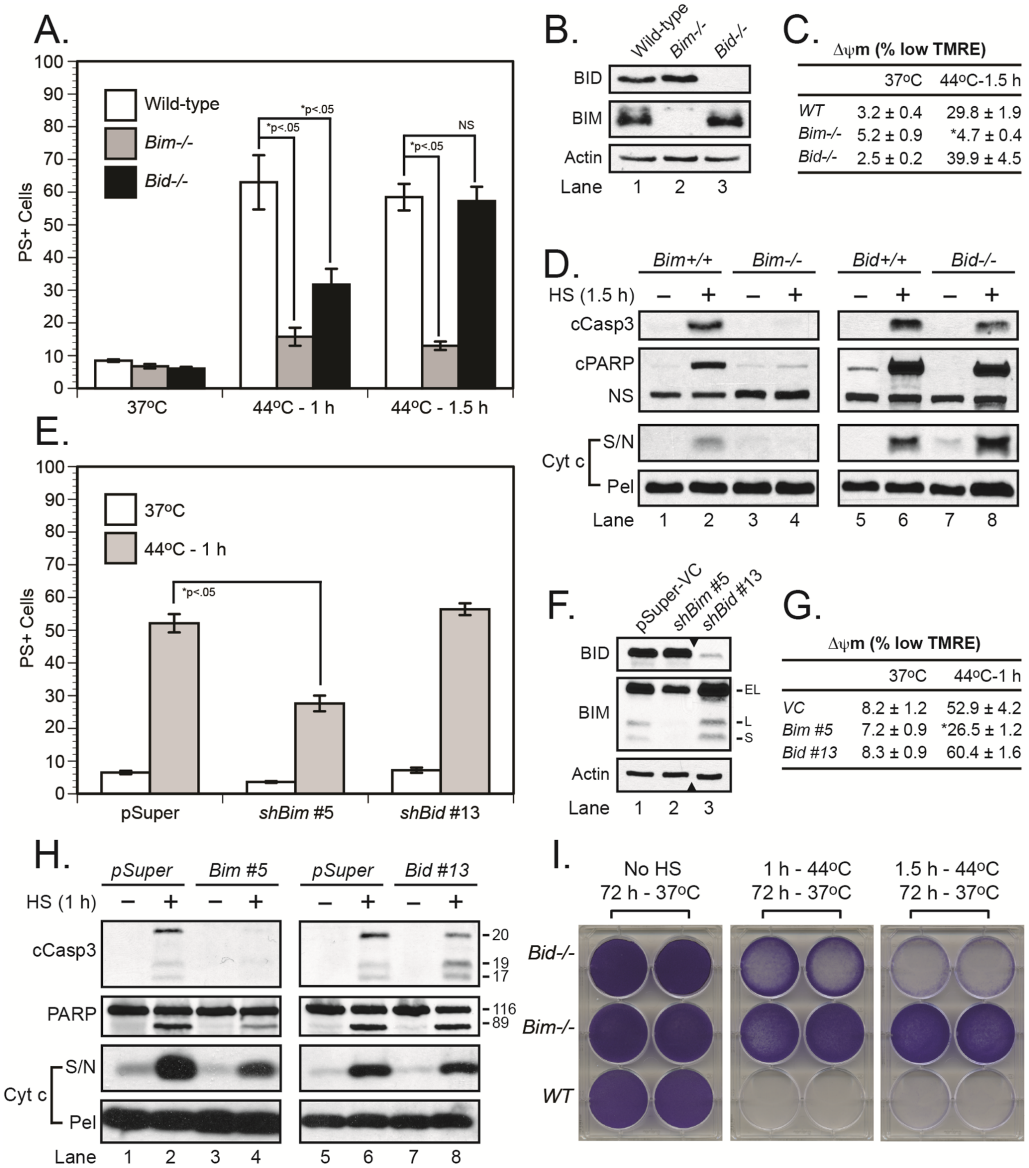
BIM is critical for heat shock-induced apoptosis. (***A-D***) Wild-type, *Bim^−/−^*, and *Bid^−/−^* MEFs (*panel B*) were exposed to heat shock (44°C for 1–1.5 h) in a humidified incubator (5% CO_2_–95% air). The cells were then transferred to a 37°C incubator and later collected for MOMP (16 h), caspase-3 activation, PARP cleavage (24 h, *panel D*), Δψm (24 h, *panel C*) and cell death measurements (24 h, *panel A*). *p<0.05, significantly different from wild-type cells. (***E-H***) Human Jurkat T cells, stably depleted of *BIM* or *BID* by RNAi (*panel F; note: black triangles denote crop marks*), were exposed to heat shock (44°C for 1 h) in a humidified incubator (5% CO_2_–95% air). As before, the cells were then transferred to a 37°C incubator and later collected for MOMP, caspase-3 activation, PARP cleavage (24 h, *panel H*), Δψm (24 h, panel G) and cell death measurements (24 h, *panel E*). (***I***) Wild-type, *Bim^−/−^*, and *Bid^−/−^* MEFs were exposed to heat shock as described above, but were left in culture afterwards for 72 h, after which the plates were stained with crystal violet. cPARP, cleaved PARP; cCasp3, cleaved/active caspase-3; S/N, supernatant; Pel, mitochondrial pellet; *p<0.05, significantly different from wild-type cells.

Heat shock reportedly induces apoptosis through the canonical intrinsic pathway in which caspase-2 is first activated and in turn cleaves BID to initiate BAX/BAK-dependent MOMP [18], [21], [28]. However, in the course of our studies, we uncovered a critical role for the BH3-only protein BIM. Loss of BIM afforded near complete protection from cell death following a 1–1.5 h exposure to heat shock at 44°C, whereas BID-deficient cells were only partially protected following a 1 h treatment (Figure 1A and B). Consistent with these cell death measurements, only *Bim^−/−^* cells avoided MOMP (as determined by cytochrome c release), loss of mitochondrial inner membrane potential (Δψm), caspase-3 activation, and PARP cleavage (Figure 1C and 1D). Importantly, other forms of stress, including DNA damage (etoposide) and ER stress (tunicamycin), readily induced apoptosis in *Bim^−/−^* MEFs (Figure S1A). Thus, collectively, the data indicated that BIM played a specific and apparently dominant role in regulating heat shock-induced apoptosis.

Previous efforts to generate stable BIM-expressing cell lines have been unsuccessful [29], and despite repeated attempts, we too were unable to stably reintroduce *Bim* into the *Bim^−/−^* MEFs. Therefore, to confirm BIM's role in heat shock-induced killing, we generated a stable human Jurkat cell line expressing a short-hairpin RNA to *Bim*. RNA interference resulted in complete loss of expression for the BIM_L_ and BIM_S_ isoforms, but only partially depleted (∼50%) the BIM_EL_ isoform (Figure 1F, lane 2). Using an optimal exposure for Jurkat cells (44°C for 1 h), we observed once again that BIM-deficient cells were resistant to cell death (Figure 1E), which correlated with the extent of total BIM knockdown, as well as the degree of MOMP, loss of Δψm, caspase-3 activation, and PARP cleavage (Figure 1E-H). A previously characterized BID-deficient clone expressed slightly higher levels of all three BIM isoforms (Figure 1F, lane 3), and as expected, it was resistant to Fas-induced apoptosis (Figure S1B) but not to heat shock-induced cell death (Figure 1E-H) [30].

Finally, even though BIM appeared to be critical for short-term protection against heat shock, we questioned whether loss of BIM could provide long-term protection. Therefore, we heat-shocked wild-type, *Bim^−/−^*, and *Bid^−/−^* MEFs for 1–1.5 h and monitored their death/growth up to 72 h. As shown in Figure 1I, *Bid^−/−^* cells were partially protected following a 1 h heat shock, but only *Bim^−/−^* cells were protected from the more extreme 1.5 h exposure.

### Caspase-2 induces cell death independently of BIM

**Figure 2:**
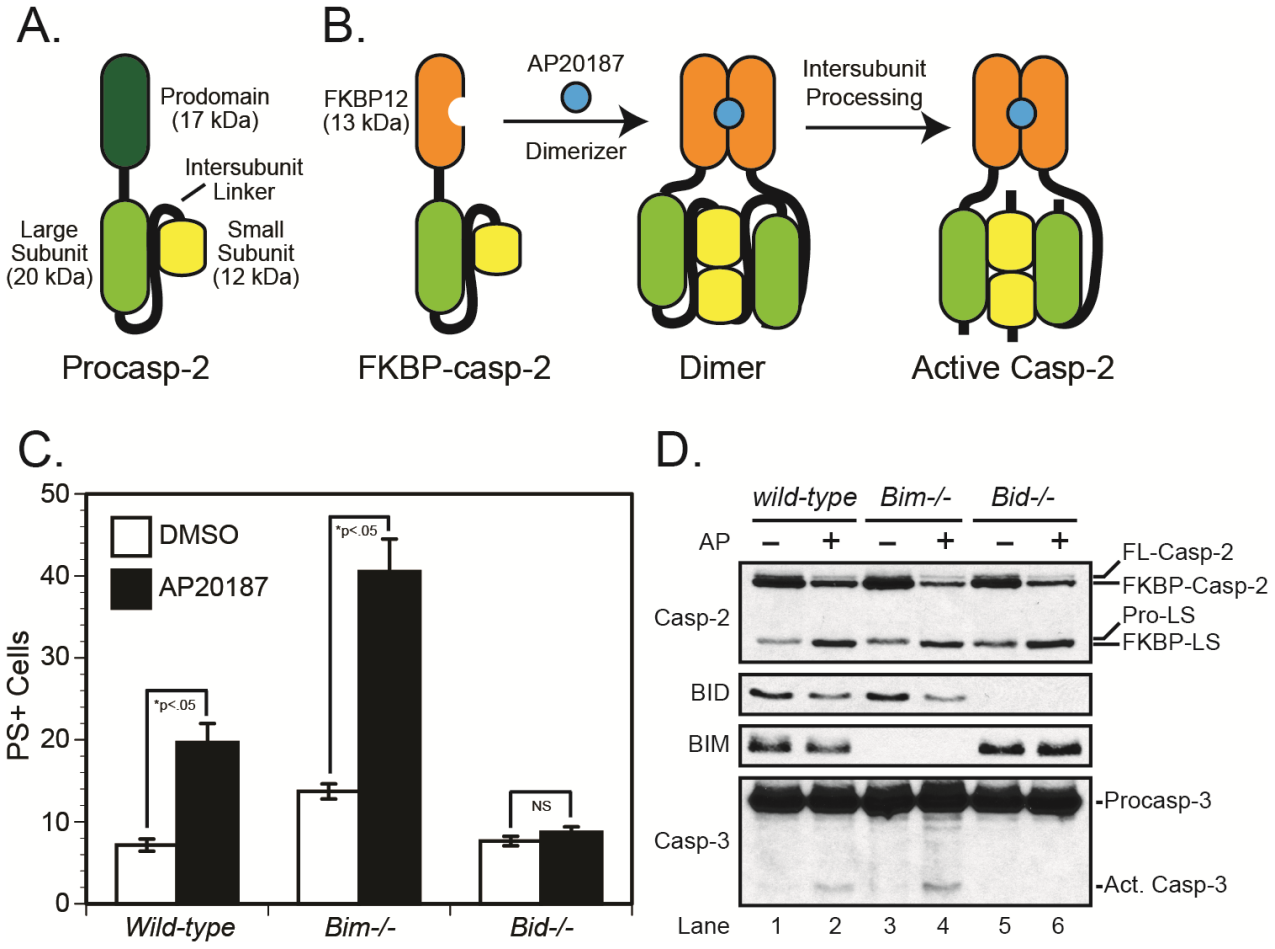
Caspase-2 induces apoptosis in a BID-dependent but BIM-independent manner. (***A and B***) Cartoons depicting wild-type caspase-2 and the FKBP-Δpro-caspase-2 fusion, in which the prodomain of caspase-2 is replaced with an FKBP protein that dimerizes upon the addition of AP20187. (***C and D***) Wild-type, *Bim^−/−^*, and *Bid^−/−^* MEFs were infected with a retrovirus expressing the FKBP-Δpro-caspase-2 fusion protein. The cells were then sorted by flow cytometry for GFP (marker)-positive cells, and the cell pools incubated in the presence or absence of AP20187 for 48 h, after which they were assayed for cell death (*panel C*) as well as activation of caspases-2 and -3 and BID cleavage where relevant (*panel D*). *p<0.05, cells treated with AP20187 were significantly different from those treated with DMSO.

Since heat shock activates the apical caspase-2 in the canonical cell death pathway, we questioned whether caspase-2 might mediate cell death *via* BIM, as has been shown for BID [15], [18]. In order to selectively activate caspase-2 we generated an FKBP-Δpro-caspase-2 construct, similar to that previously reported for caspase-9 [31] in which the prodomain of caspase-2 was replaced with a modified FKBP protein that can be induced to dimerize upon exposure to the chemical ligand AP20187 (Figure 2A and B). Since dimerization is thought to mediate the activation of initiator caspases, including caspase-2, we retrovirally-expressed FKBP-Δpro-caspase-2 in wild-type, *Bim^−/−^*, and *Bid^−/−^* MEFs and sorted the cells using a GFP marker. Following exposure to AP20187, we observed activation of caspase-2 in each of the three isolated cell pools, and as previously reported [18], the *Bid^−/−^* cells were resistant to caspase-3 activation and cell death (Figure 2C and D). By contrast, both the wild-type and *Bim^−/−^* cells displayed BID cleavage, caspase-3 activation, and cell death, indicating that BIM is not essential for caspase-2-mediated cell death. In fact, for reasons that remain unclear, *Bim-/- cells* were even more sensitive than wild-type cells to caspase-2-mediated cell death, despite being entirely resistant to heat shock-induced death.

### Heat shock induces cell death through a BAX/BAK-dependent pathway

**Figure 3:**
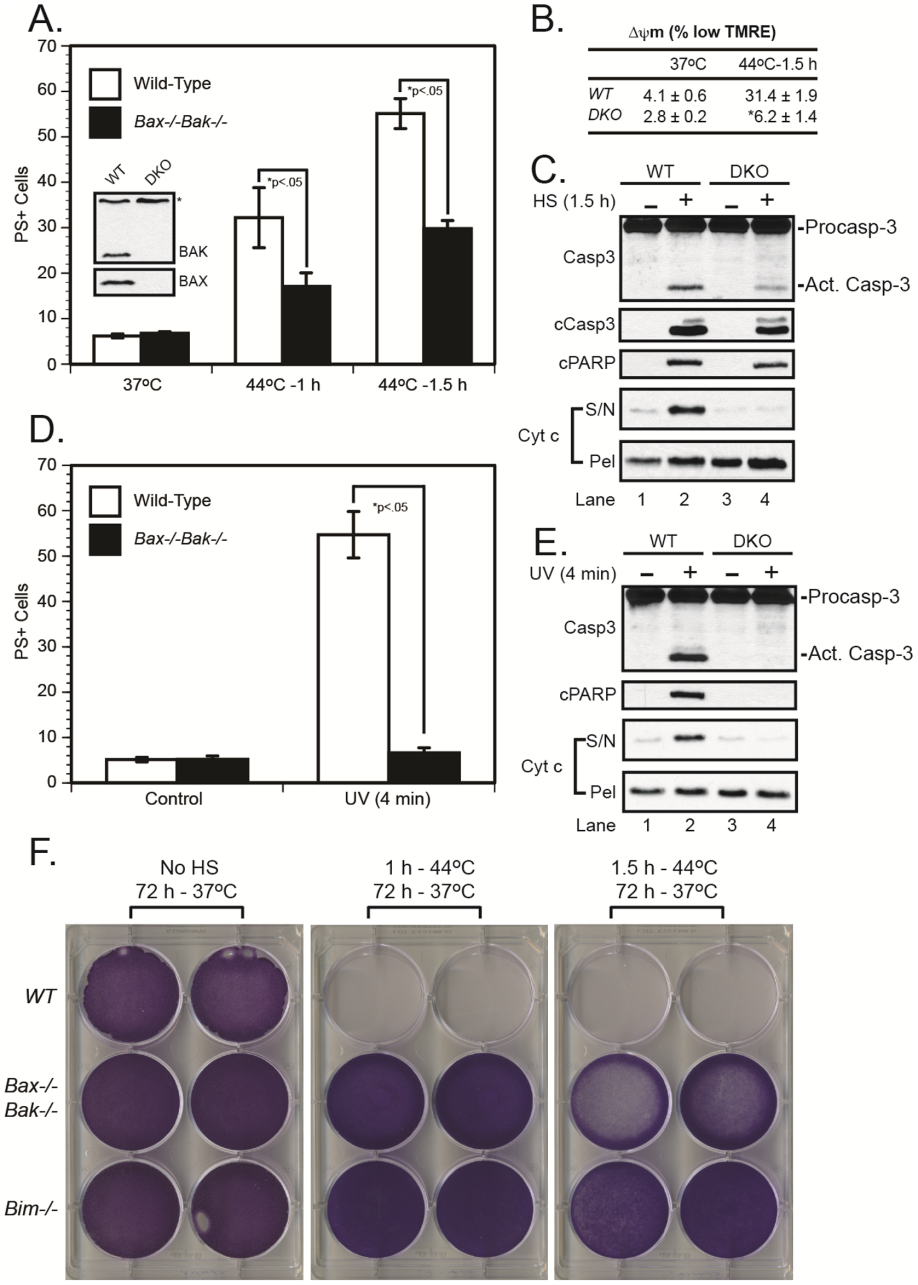
*Bax^−/−^Bak^−/−^* cells resist heat shock-induced MOMP and loss of Δψm, but still undergo caspase-3 activation and cell death. Wild-type and *Bax^−/−^Bak^−/−^* DKO cells were exposed to (*A-C*) heat shock (44°C for 1–1.5 h) in a humidified incubator (5% CO_2_–95% air), or (*D and E*) UV irradiation (4 min) on a transilluminator. The cells were then transferred to a 37°C incubator and later collected for MOMP (16 h), caspase-3 activation, PARP cleavage (24 h, *panels C and E*), Δψm (24 h, *panel B*) and cell death measurements (24 h, *panels A and D*). cPARP, cleaved PARP; cCasp3, cleaved/active caspase-3; S/N, supernatant; Pel, mitochondrial pellet; *p<0.05, significantly different from wild-type cells.

Since BIM played a critical role in heat shock-induced cell death, we expected that it was likely to induce MOMP and cell death through its activation of the multidomain pro-apoptotic BCL-2 family members, BAX and/or BAK. To our surprise, however, while loss of BAX and BAK did protect cells from heat shock-induced death, ∼30% of cells still died regardless of BAX/BAK expression (Figure 3A-C). Notably, the *Bax^−/−^Bak^−/−^* cells remained entirely resistant to UV-induced apoptosis (Figure 3D and E), as well as DNA damage and ER stress-induced cell death (Figure S2). Remarkably, the *Bax^−/−^Bak^−/−^* cells failed to undergo MOMP or loss in Δψm following heat shock, but nevertheless activated caspase-3 and cleaved PARP, albeit to a lesser extent (Figure 3B; 3C, lanes 2 and 4). Despite the unexpected caspase activation and cell death in the *Bax^−/−^Bak^−/−^* cells, those that were alive at 24 h remained viable and populated the culture dish by 72 h (Figure 3F).

**Figure 4:**
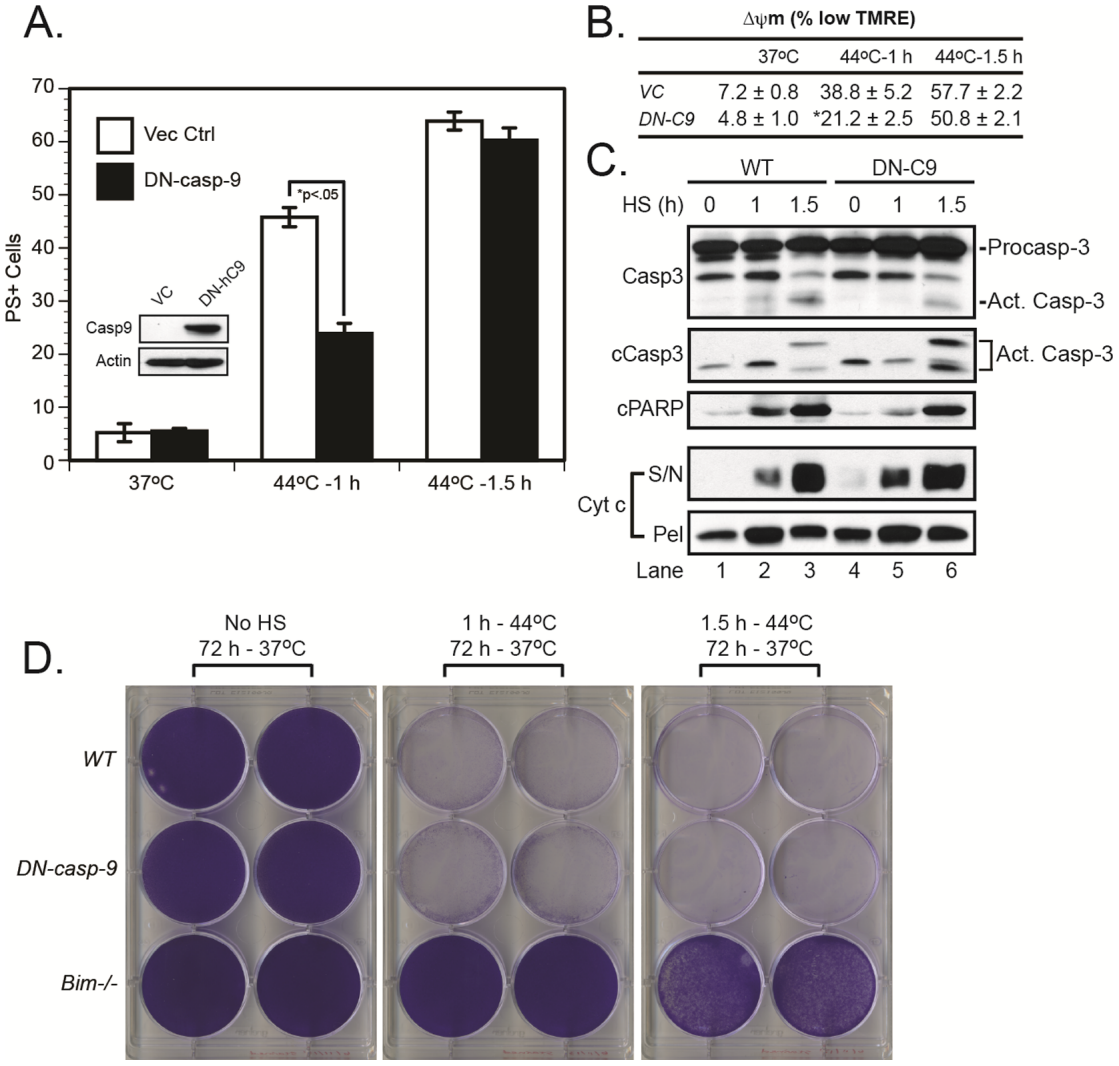
Inhibition of apoptosome-dependent caspase-9 activity weakly inhibits heat shock-induced apoptosis but does not provide long-term protection. (*A-C*) Vector control MEFs and those expressing a DN-caspase-9 (C287A) were exposed to heat shock (44°C for 1–1.5 h) in a humidified incubator (5% CO_2_–95% air). The cells were then transferred to a 37°C incubator and later collected for MOMP (16 h), caspase-3 activation, PARP cleavage (24 h, *panel C*), Δψm (24 h, *panel B*) and cell death measurements (24 h, *panel A*). (*D*) Wild-type, *Bim^−/−^*, *and DN-caspase-9* MEFs were exposed to heat shock as described above, but were left in culture afterwards for 72 h, after which the plates were stained with crystal violet. cPARP, cleaved PARP; cCasp3, cleaved/active caspase-3; S/N, supernatant; Pel, mitochondrial pellet; *p<0.05, significantly different from vector control cells.

Finally, to determine the importance of the apoptosome, downstream of MOMP, we sought to inhibit the complex through overexpression of a dominant-negative caspase-9 (DN-caspase-9). While DN-caspase-9 expression partially inhibited cell death following exposure to heat shock (1 h), it failed to inhibit cell death following a longer 1.5 h exposure and provided no long-term protection (Figure 4A-D), consistent with our previous results in *caspase-9^−/−^* MEFs [32]. It is interesting to note that cells deficient in Apaf-1 appear to be more resistant to heat shock than those deficient in caspase-9 [30], [32], implying that Apaf-1 may play a role in the heat shock response that is independent of the apoptosome. In any event, *Bax^−/−^Bak^−/−^* cells were slightly inferior to *Bim^−/−^* cells with regard to long-term survival, but they were clearly more resistant to cell death compared with wild-type, *Bid^−/−^*, or *DN-caspase-9* cells. Thus, the data indicated that, following heat shock, BIM induced significant cell death through a BAX/BAK-dependent pathway, consistent with its well-established role as a direct activator of BAX and BAK [6]. However, the fact that heat shock triggered some caspase-3 activation in *Bax^−/−^Bak^−/−^* cells in the absence of MOMP, but failed to do so in *Bim^−/−^* cells, suggests that BIM may be capable of activating caspase-3 in a BAX/BAK-independent manner. A BIM mutant lacking its BH3 domain has been shown to suppress L929 fibroblast colony formation *in vitro* [33]; however, attempts to significantly deplete *Bax^−/−^Bak^−/−^* MEFs of *Bim* were unsuccessful. Thus, at this time, we cannot confirm that BIM is responsible for the heat shock-induced caspase-3 activation and cell death observed in the *Bax^−/−^Bak^−/−^* MEFs.

### Loss of MCL-1 or antagonism of BCL-2/BCL-X_L_ sensitizes cells to heat shock-induced apoptosis

**Figure 5:**
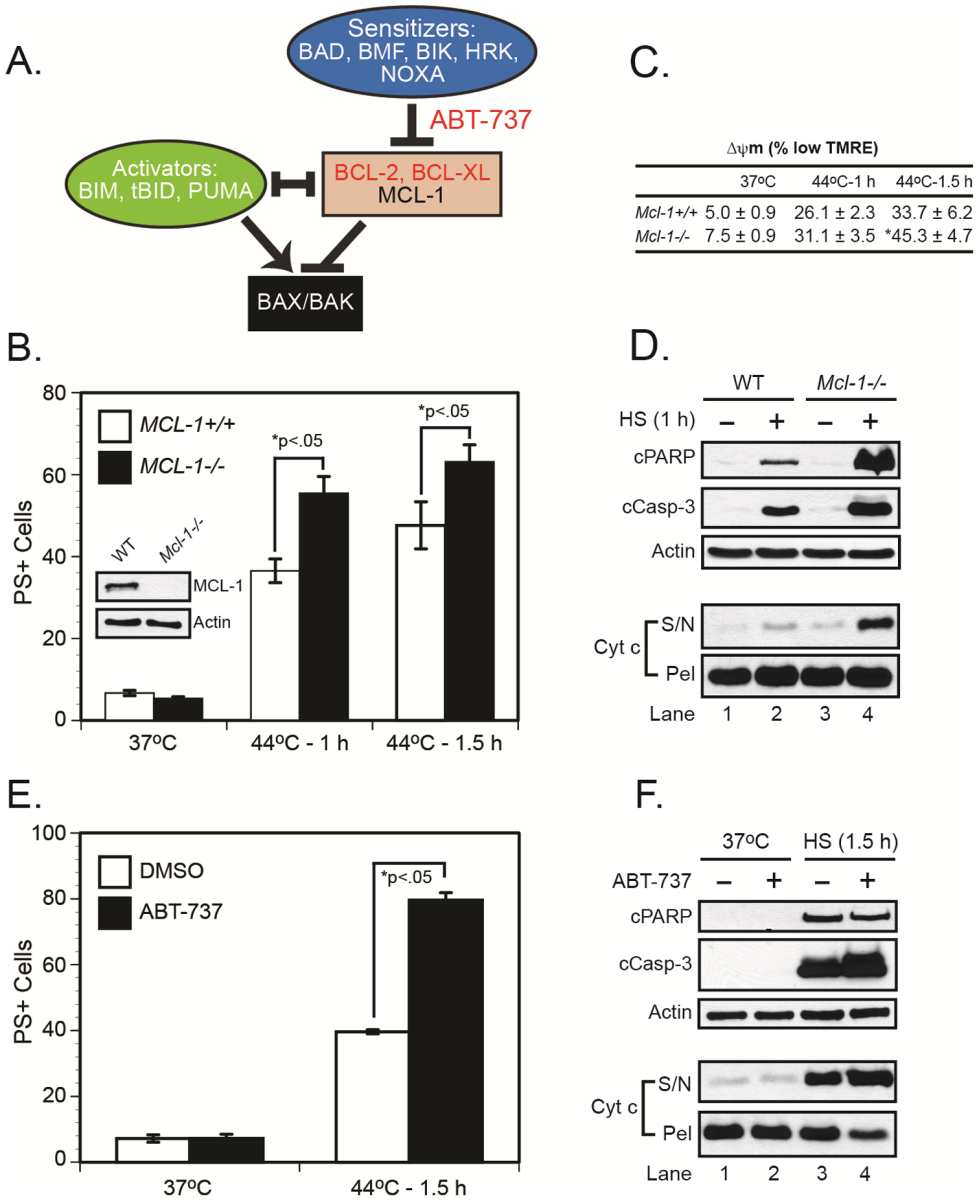
Loss of MCL-1 or inhibition of BCL-2/BCL-X_L_ potentiates heat shock-induced apoptosis. (***A***) Cartoon illustrating the interactions of BH3-only proteins with multidomain proapoptotic (BAX and BAK) and antiapoptotic (MCL-1, BCL-2, and BCL-X_L_) BCL-2 family members. BH3-only proteins can function as BAX/BAK activators (BIM, tBID, PUMA), or as sensitizers (NOXA, BAD, BMF, BIK, and HRK) that displace activators from antiapoptotic family members. (***B-D***) Wild-type and *Mcl-1^−/−^* MEFs (*panel B, inset*) were exposed to heat shock (44°C for 1–1.5 h) in a humidified incubator (5% CO_2_–95% air). The cells were then transferred to a 37°C incubator and later collected for MOMP (16 h), caspase-3 activation, PARP cleavage (24 h, *panel D*), Δψm (24 h, *panel C*) and cell death measurements (24 h, *panel B*). *p<0.05, significantly different from wild-type cells. (***E and F***) Wild-type MEFs were preincubated with DMSO (control) or ABT-737 (250 nM) for 1 h and subsequently exposed to heat shock (44°C for 1.5 h) in a humidified incubator (5% CO_2_–95% air). The cells were then transferred to a 37°C incubator and later collected for MOMP (16 h), caspase-3 activation, PARP cleavage (24 h, *panel F*) and cell death measurements (24 h, *panel E*). *p<0.05, cells treated with ABT-737 were significantly different from those treated with DMSO. cPARP, cleaved PARP; cCasp3, cleaved/active caspase-3; S/N, supernatant; Pel, mitochondrial pellet.

Previous studies indicate that BIM, BID, and PUMA function as *direct activators* of BAX and BAK, whereas the remaining BH3-only family members function as *sensitizers*, *i.e.* they cannot directly activate BAX and BAK, but they can neutralize the antiapoptotic family members (BCL-2, BCL-X_L_, MCL-1) that inhibit direct activators (Figure 5A) [9]. Mosser and colleagues have recently reported that MCL-1 plays an important role in suppressing heat shock-induced apoptosis [34]. Consistent with their results, loss of MCL-1 sensitized cells to heat shock-induced MOMP, loss of Δψm, caspase-3 activation, PARP cleavage, and cell death (Figure 5B-D). However, the BCL-2/BCL-X_L_ antagonist, ABT-737, also sensitized MEFs to heat shock-induced cell death (Figure 4E and F). Since ABT-737 functions as a sensitizer and BIM is critical for heat shock-induced killing, the simplest explanation is that ABT-737 most likely liberated BIM from BCL-2 or BCL-X_L_, which in turn activated BAX and/or BAK. This interpretation is also consistent with our previous data in which BCL-2 overexpression protected BAX-deficient Jurkat T cells from heat shock-induced apoptosis [32], even though BCL-2 does not inhibit BAK [11].

## Discussion

**Figure 6:**
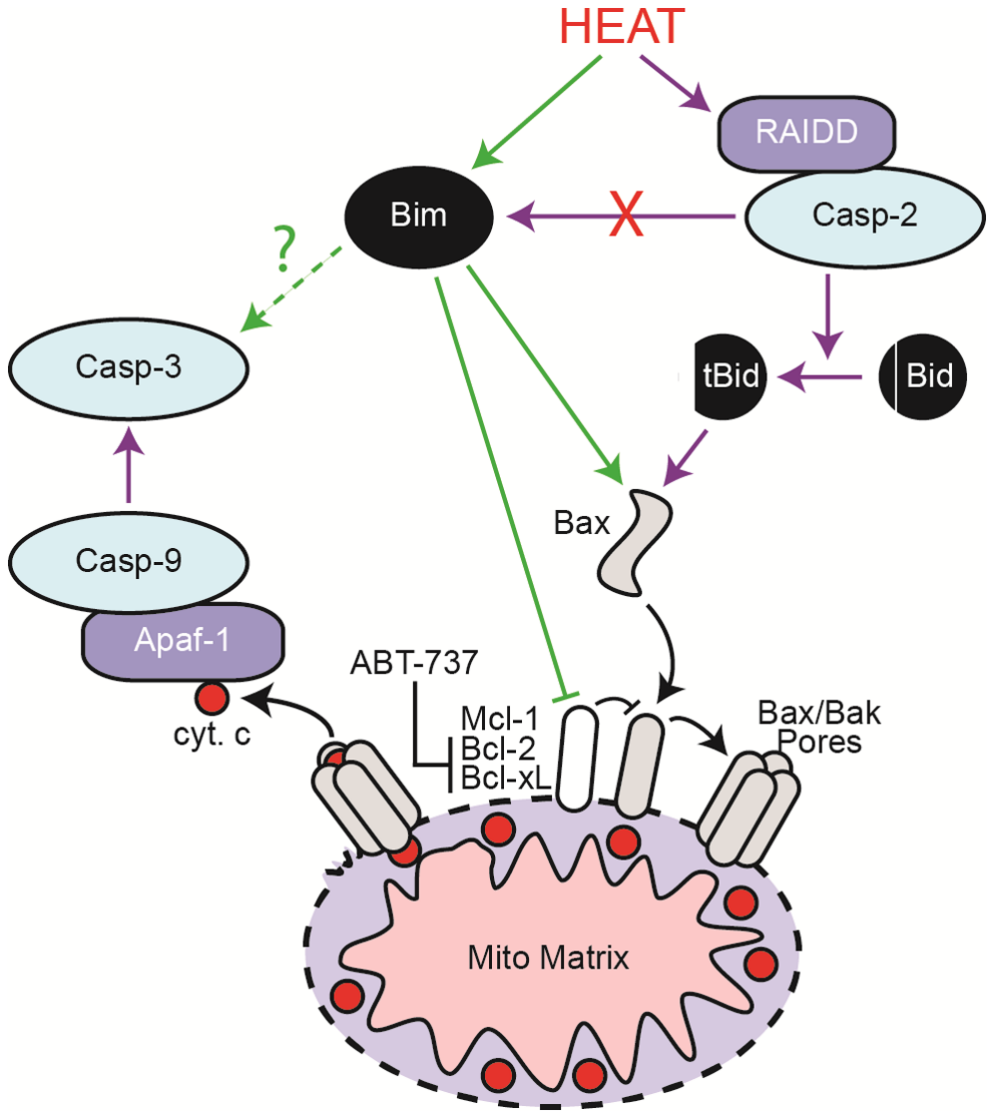
Model of BIM-induced cell death following heat shock. According to the canonical pathway, heat shock induces formation of a RAIDD-caspase-2 complex that activates caspase-2. Following cleavage by caspase-2, truncated Bid (tBID) then stimulates BAX/BAK-dependent MOMP, cytochrome c release, and formation of the Apaf-1/caspase-9 apoptosome complex, which in turn activates the effector caspase-3. In our hands, however, loss of BID or inhibition of the apoptosome provides only modest short-term protection at lower exposures (1 h, 44°C), whereas loss of BIM profoundly inhibits cell death and facilitates long-term protection. In the context of heat shock, BIM induces MOMP and loss of Δψm in a BAX/BAK-dependent manner (and may be responsible for triggering caspase-3 activation in the absence of detectable mitochondrial injury). Thus, BIM appears to mediate an alternative (and perhaps dominant) pathway to heat shock-induced apoptosis.

The ability to adapt and survive heat shock is fundamentally important for cellular life. Heat shock induces various cellular disturbances, including defects in DNA repair, cell cycle arrest, and cytoskeletal damage. Results from hundreds of studies have characterized survival pathways initiated by heat shock, including the upregulation of heat shock proteins that function as chaperones in the repair of misfolded proteins [35]. In some cases, however, cells are unable to overcome the damage induced by heat shock and instead initiate a cell death program. There is significant interest in understanding better the underlying mechanisms that mediate heat shock-induced apoptosis, since heat shock preferentially targets malignant cells and cancers for reasons that remain unclear. Most data suggest that heat shock kills cells through activation of the intrinsic apoptosis pathway, and several recent papers from Green and colleagues indicate that heat shock stimulates selective formation of a cytoplasmic RAIDD-caspase-2 complex, which in turn activates caspase-2 and cleaves BID [18], [21], [28]. tBID then stimulates MOMP, cytochrome c release, and formation of the Apaf-1-caspase-9 apoptosome (Figure 6) [18], [21]. Green and colleagues also provide evidence that heat itself induces conformational changes in BAX and/or BAK that render them more susceptible to activation by tBID [36].

Additional reports, however, suggest this canonical pathway may not fully account for the cell death induced by heat shock. In some instances, caspase-2 and BID are not essential for cell death or serve as an amplification loop to promote MOMP and caspase activation downstream of the apoptosome [30], [32]. In the current studies, we have discovered that another BH3-only family member, BIM, plays a significant role in mediating cell death, independently of the caspase-2-BID pathway. By assessing the survival of *Bim^−/−^*, *Bid^−/−^*, *Bax^−/−^Bak^−/−^*, and *DN-caspase-9* cells at different exposure levels, we found that loss of BID afforded some early protection to “low-dose” heat shock, but failed to provide short or long-term protection following a “high-dose” exposure. By comparison, loss of BIM afforded significant protection at both doses, apparently even exceeding the protection observed in *Bax^−/−^Bak^−/−^* cells. Indeed, unlike *Bim^−/−^* cells, Bax/Bak-deficient cells underwent reduced but significant caspase-3 activation, PARP cleavage, and cell death. Collectively, these experiments indicate that at a minimum BIM induces apoptosis following heat shock through a BAX/BAK-dependent pathway, consistent with its established role as a direct activator of BAX and BAK [6]. Whether BIM also induces BAX/BAK-independent activation of caspase-3, in the absence of MOMP, remains unclear. As to how BIM is activated following heat shock, it is worth noting that heat shock disrupts intermediary, actin, and tubulin networks [35], and BIM, which associates with the LC8 chain of the dynein motor complex, is liberated in response to cytoskeletal damage [33]. Moreover, heat shock is a strong activator of c-Jun N-terminal kinases (JNKs) [37], and BIM_EL_ contains a JNK phosphorylation site at Thr112, which disrupts its association with LC8 [38].

Given that *Bim^−/−^* cells were more resistant to heat shock than *Bid^−/−^* cells, it is tempting to conclude that the BIM-mediated apoptosis pathway is dominant and that the caspase-2-BID pathway represents an amplification loop. However, we remain somewhat skeptical of this interpretation, given that caspase-2 associates with RAIDD in cells following heat shock [28] and that adapter proteins typically interact only with apical caspases to initiate a caspase cascade [39]. Thus, in our view, the caspase-2-BID pathway likely represents an alternative pathway that is most active at lower temperatures or shorter exposures. Since active caspase-2 does not require BIM in order to kill cells, the BIM and caspase-2-BID pathways appear to function independently of one another, and these may be purposefully redundant pathways to ensure that severely heat-shocked tissues do not survive.

## Materials and Methods

### Antibodies and reagents

The following antibodies were purchased from Cell Signaling Technology (Danvers, MA): BAX (CS 2772); BAK (CS 3814); BID (CS 2002), cleaved caspase-3 (CS 9662); total caspase-3 (CS 8G10); hCaspase-9 (CS 9502); β-actin (CS 4970); cytochrome c (CS 11940); PARP (CS 9542); and cleaved PARP (CS 9544). Other antibodies used were as follows: BIM (cat. No. ADI-AAP-330, Enzo Life Sci., Farmingdale, NY); MCL-1 (cat. No. 600-401-394S, Rockland Immunochemicals, Inc., Gilbertsville, PA); and caspase-2 (cat. No. MAB3501, Chemicon/EMD Millipore, Billerica, MA). ABT-737 (cat. No. S1002) was purchased from Selleckchem (Houston, TX). The AP20187 homodimerizer (cat. No. 635060) was purchased from Clontech (Mountainview, CA). Digitonin (D141) was obtained from Sigma (St. Louis, MO). Fetal Bovine Serum was obtained from Atlanta Biologicals (Lawrenceville, GA), and DMEM and RPMI were purchased from Corning Cellgro (Manassas, VA).

### Plasmids

The following sense strand sequence was used to silence human *Bim*: 5′-GACCGAGAAGGUAGACAAUUGdTdT-3′ [40] and was cloned into the pSuper/Neo vector. Jurkat cells were then electroporated with 10 µg of either empty vector or pSuper/*shBim*, and after 24 h, live cells were separated on a Ficoll density gradient by centrifugation. Stable clones were then selected in G418 (1 mg/mL), and knockdown levels determined by western blotting. A previously published pSuper/*shBid* clone was similarly generated [30]. To generate the N-terminal fusion of the dimerization domain of human FK506 binding protein (FKBP1A) with CARD-deleted caspase-2 (*i.e.* pFKBP-Δpro-caspase-2), the *Bam*HI and *Eco*RI sites in the human caspase-2 sequence were first mutated by site-directed mutagenesis using the following primers: C2_BHI_US: 5′-GAACCACGCAGGGTCCCCTGGGTGCG-3′; C2_BHI_DS: 5′-CGCACCCAGGGGACCCTGCGTGGTTC-3′; C2_ECORI_US: 5′-CTCCTGGCACAGAGTTCCACCGGTGCA-3′; C2_ECORI_DS: 5′-TGCACCGGTGGAACTCTGTGCCAGGAG-3′. The Δpro-caspase-2 sequence was then amplified using the following primers: BglII_C2LS_US (from amino acid 170) 5′-AGAAGATCTGGTCCTGTCTGCCTTCAGGTG-3′; EcoNot_C2SS_DS 5′-GAAGAATTCGCGGCCGCTCATGTGGGAGGGTGTCCTGG-3′. The PCR products were digested with *Bgl*II/*Eco*RI and cloned into pMSCV-FKBP-IRES-GFP. DN-caspase-9 was generated as previously reported [41]. The accuracy of all constructs was confirmed by sequencing.

### Cell culture and transfections

MEFs were grown in DMEM supplemented with 10% FBS (Atlanta Biologicals, Lawrenceville, GA), 1% penicillin–streptomycin (100 units/mL), and 2 mM glutamine. Jurkat cells were maintained in RMPI 1640 supplemented with 10% FBS, 1% penicillin–streptomycin (100 units/mL), and 1 mM glutamine. Cells were maintained at 37°C in humidified air containing 5% CO_2_ and were routinely passaged every 2 d. *Bim^−/−^* and *Bid^−/−^* MEFs were a kind gift from Dr. David C. S. Huang (Walter and Eliza Hall Institute of Medical Research, Melbourne, Australia) [12]. *Bax^−/−^Bak^−/−^* MEFs were a kind gift from Dr. Craig B. Thompson (Memorial Sloan–Kettering Cancer Center, NY) [42]. *Mcl-1^−/−^* MEFs were a kind gift from Dr. Joseph T. Opferman (St. Jude Children's Research Hospital, Memphis, TN) [43].

For the generation of MEFs expressing inducible FKBP-Δpro-caspase-2, GP2-293 cells were transfected with 6 µg each of the pAmphotropic receptor and pFKBP/Δpro-caspase-2 plasmids using Lipofectamine®2000, according to the manufacturer's recommendations (Invitrogen). After a 48 h transfection, the viral supernatant was mixed with polybrene (7 µg/mL) and exposed to MEFs (30% confluent) for 4 h. After infection, cells were expanded for 3 d and then cell-sorted for GFP-positive cells on a BD FACS-ARIA. The isolated cell pools were then analyzed by immunoblotting for the expression of FKBP-Δpro-caspase-2 fusion and GFP. For the dimerization experiments, 150×10^3^ cells/well were seeded into 12-well plates and 18 h later the AP20187 homodimerizer (100 nM) was added. The uptake of propidium iodide (PI) was then quantified 48 h later by flow cytometry.

For generation of the stable cell line expressing DN-caspase-9, human procaspase-9 (C287A) was cloned into the FG9 lentiviral plasmid and cotransfected with pRRE, pHCMV and pRSV-Rev into HEK293T cells, using TransIT®-2020 according to manufacturer's instructions (Mirus®). Forty-eight hours later, viral supernatant was obtained and mixed with polybrene (7 µg/ml), filtered through a 0.45 µM filter (Millipore), and added to wild-type MEFs. The infected MEFs were subsequently expanded and subjected to hygromycin selection (200 µg/ml) for 7 d, after which DN-caspase-9 expression was detected by immunoblotting for human caspase-9.

### Heat shock treatments

Cells were plated at 0.3−0.5×10^6^ cells/well in 6-well plates 20 h prior to heat shock. Exposures were done in a tissue culture incubator at 44°C with 5% CO_2_ for various periods of time, after which the cells were returned to a 37°C incubator for “recovery”. Samples were collected for analyses at various time points post-heat shock. To examine long-term survival, cells were prepared and treated as above, except that fresh media was added to the cells after 24 h and the plates were cultured for an additional 48 h at 37°C. At 72 h post-heat shock, the cells were fixed with 70% EtOH for 10 min, stained with crystal violet for 45 min, washed with tap water, and allowed to air dry prior to image analysis [44].

### Cell death and *Δ*ψm assays

Trypsinized MEFs or Jurkat T cells (1×10^6^) were pelleted at 400 x *g* for 4 min, washed with PBS, and resuspended in 1 mL of Annexin V binding buffer (10 mM HEPES, pH 7.4, 140 mM NaCl, 2.5 mM CaCl_2_). Cells were then incubated with 100 ng/mL Annexin-FITC for 8 min, and propidium iodide was added just prior to flow cytometric analysis. Recombinant Annexin V was expressed and purified in-house, labeled with FITC, and dialyzed to remove unconjugated dye. Cell populations, labeled with FITC and/or PI, were analyzed by flow cytometry (Beckman-Coulter, Fullerton, CA, USA). Similarly, to assess the loss in Δψm, suspensions containing 1×10^6^ MEFs or Jurkat cells were incubated at 37°C for 20 min in pre-warmed (37°C) media containing 100 nM tetramethylrhodamine (TMRE; Molecular Probes). Cells were then washed twice with PBS and analyzed by flow cytometry.

### Western blotting

Cell pellets were lysed in RIPA lysis buffer on ice for 20 min, spun at 18,000 x *g* for 10 min at 4°C, and the supernatants normalized for protein concentration by the Bradford assay. Equal amounts of protein were resolved by SDS-PAGE, transferred to nitrocellulose membranes, and blocked in 0.1% Tween-TBS with 5% non-fat milk prior to incubation with primary antibodies (1∶1,000–2,000).

### Cytochrome c release assay

16 h post-treatment, 5×10^6^ cells were trypsinized (when necessary), washed twice in PBS, and permeabilized in MOMP lysis buffer (20 mM HEPES, pH 7.4, 250 mM sucrose, 1 mM EDTA, 75 mM KCl, 2.5 mM MgCl_2_) containing 0.05% digitonin (always added fresh) on ice for 5 min. The cells were then centrifuged at 15,000 x *g* for 10 min at 4°C to collect the “cytosolic fractions”. The pellets were lysed in RIPA buffer, as described above, to obtain the “mitochondrial fractions”. Jurkat cells were similarly permeabilized utilizing 0.02% digitonin. The protein concentrations of cytosolic and mitochondrial fractions were measured by the Bradford assay and resolved by SDS-PAGE.

### Statistics

All experiments were performed at least three times. Each data point represents the mean ± S.E.M. Multiple group comparisons were performed by one-way ANOVA, followed by a Tukey post hoc analysis (p<0.05 was considered statistically significant).

## Supporting Information

Figure S1 ***Bim^−/−^*** **MEFs and BID-deficient Jurkat cells respond normally to other proapoptotic stimuli.** (***A***) Wild-type and *Bim^−/−^* MEFs were exposed to heat shock (44°C for 1–1.5 h) in a humidified incubator (5% CO2–95% air), or treated with etoposide (5 µM) or tunicamycin (0.25 µg/mL), and subsequently assayed for cell death at 24 h. (***B***) Jurkat T cells stably, expressing a scrambled or *Bid* shRNA, were exposed to agonistic human CD95 antibody (CH11; 100–250 ng/mL) for 4 h and subsequently assayed for cell death by Annexin V-PI staining and flow cytometry.
(TIFF)

Figure S2 ***Bax^−/−^Bak^−/−^*** **MEFs respond normally to other proapoptotic stimuli.** Wild-type and *Bax^−/−^Bak^−/−^* MEFs were exposed to etoposide (5 µM) or tunicamycin (0.25 µg/mL) and subsequently assayed for cell death at 24 h.
(TIFF)
