# Supplementary figures and images for: BH3-Only Protein BIM Mediates Heat Shock-Induced Apoptosis
Source: PLoS One. 2014 Jan 10;9(1):e84388. doi: 10.1371/journal.pone.0084388 (PMC3888412; doi:10.1371/journal.pone.0084388)

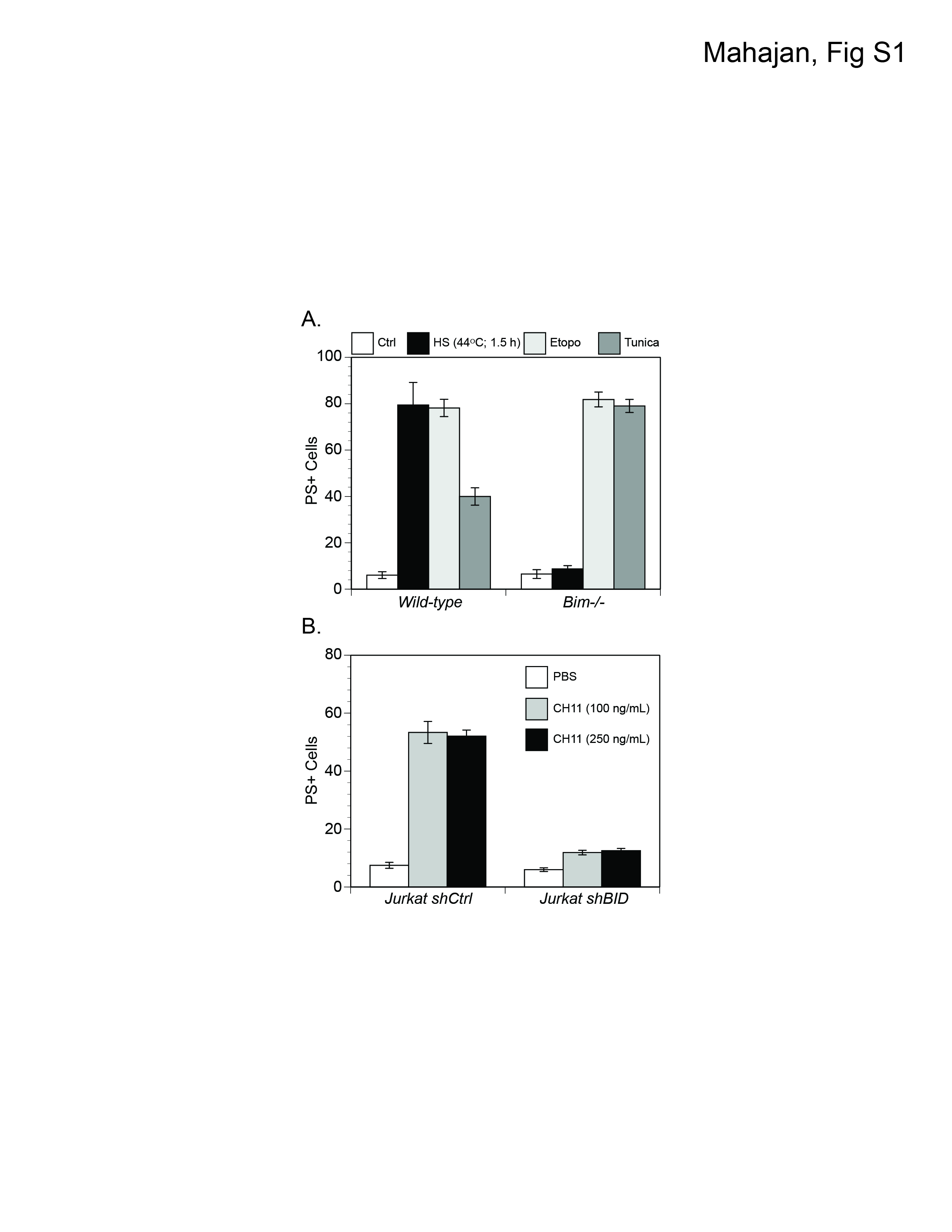

Supplement: Figure S1 — Bim−/− MEFs and BID-deficient Jurkat cells respond normally to other proapoptotic stimuli. (A) Wild-type and Bim−/− MEFs were exposed to heat shock (44°C for 1–1.5 h) in a humidified incubator (5% CO2–95% air), or treated with etoposide (5 µM) or tunicamycin (0.25 µg/mL), and subsequently assayed for cell death at 24 h. (B) Jurkat T cells stably, expressing a scrambled or Bid shRNA, were exposed to agonistic human CD95 antibody (CH11; 100–250 ng/mL) for 4 h and subsequently assayed for cell death by Annexin V-PI staining and flow cytometry. (TIFF) [file pone.0084388.s001.tiff]

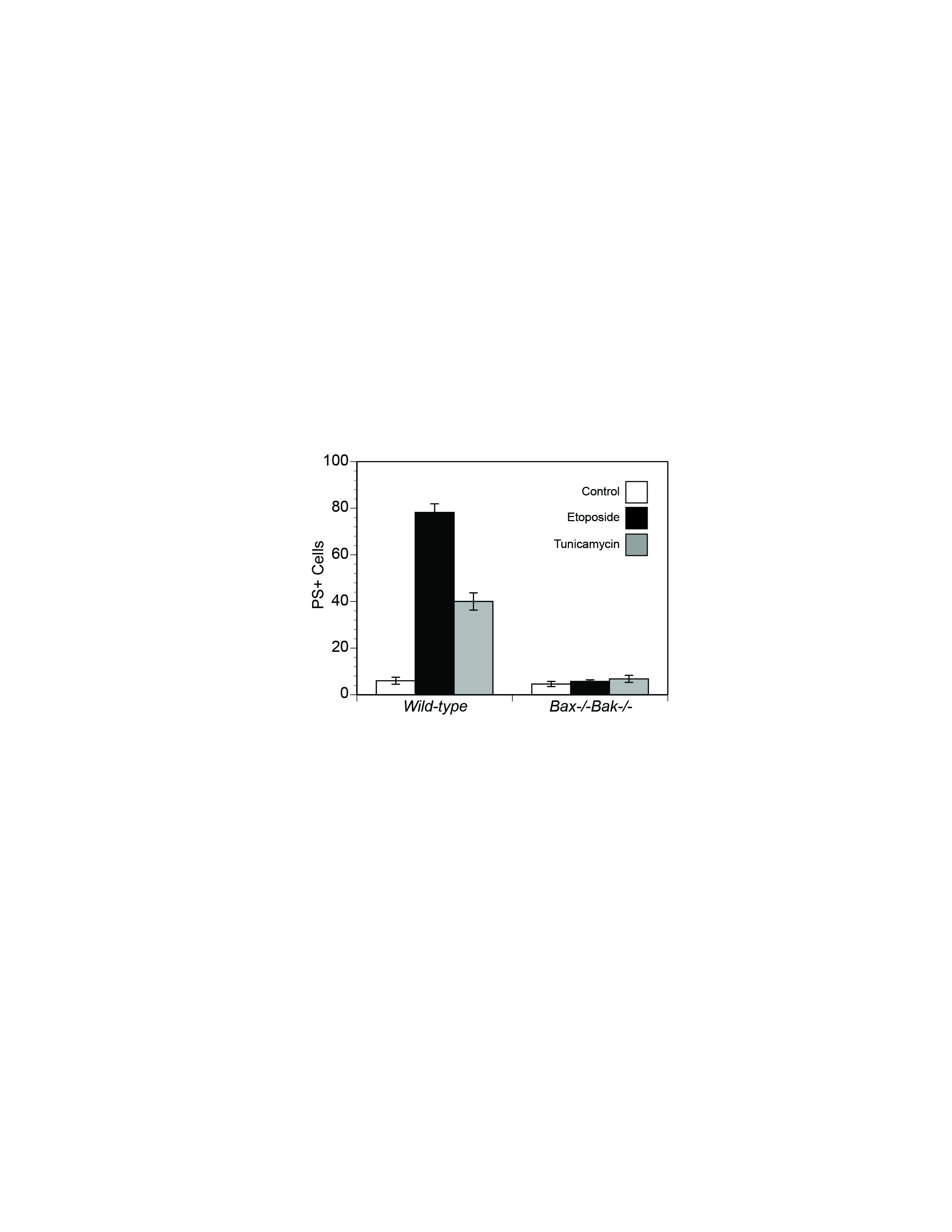

Supplement: Figure S2 — Bax−/−Bak−/− MEFs respond normally to other proapoptotic stimuli. Wild-type and Bax−/−Bak−/− MEFs were exposed to etoposide (5 µM) or tunicamycin (0.25 µg/mL) and subsequently assayed for cell death at 24 h. (TIFF) [file pone.0084388.s002.tiff]
